# Comparative analysis of missing value imputation methods to improve clustering and interpretation of microarray experiments

**DOI:** 10.1186/1471-2164-11-15

**Published:** 2010-01-07

**Authors:** Magalie Celton, Alain Malpertuy, Gaëlle Lelandais, Alexandre G de Brevern

**Affiliations:** 1 INSERM UMR-S 726, Equipe de Bioinformatique Génomique et Moléculaire (EBGM), DSIMB, Université Paris Diderot - Paris 7, 2, place Jussieu, 75005, France; 2 UMR 1083 Sciences pour l'Œnologie INRA, 2 place Viala, 34060 Montpellier cedex 1, France; 3 Atragene Informatics, 33-35, Rue Ledru-Rollin 94200 Ivry-sur-Seine, France; 4 INSERM UMR-S 665, DSIMB, Université Paris Diderot - Paris 7, Institut National de Transfusion Sanguine (INTS), 6, rue Alexandre Cabanel, 75739 Paris cedex 15, France

## Abstract

**Background:**

Microarray technologies produced large amount of data. In a previous study, we have shown the interest of *k-Nearest Neighbour *approach for restoring the missing gene expression values, and its positive impact of the gene clustering by hierarchical algorithm. Since, numerous replacement methods have been proposed to impute missing values (MVs) for microarray data. In this study, we have evaluated twelve different usable methods, and their influence on the quality of gene clustering. Interestingly we have used several datasets, both kinetic and non kinetic experiments from yeast and human.

**Results:**

We underline the excellent efficiency of approaches proposed and implemented by Bo and co-workers and especially one based on expected maximization (*EM_array*). These improvements have been observed also on the imputation of extreme values, the most difficult predictable values. We showed that the imputed MVs have still important effects on the stability of the gene clusters. The improvement on the clustering obtained by hierarchical clustering remains limited and, not sufficient to restore completely the correct gene associations. However, a common tendency can be found between the quality of the imputation method and the gene cluster stability. Even if the comparison between clustering algorithms is a complex task, we observed that *k-means *approach is more efficient to conserve gene associations.

**Conclusions:**

More than 6.000.000 independent simulations have assessed the quality of 12 imputation methods on five very different biological datasets. Important improvements have so been done since our last study. The *EM_array *approach constitutes one efficient method for restoring the missing expression gene values, with a lower estimation error level. Nonetheless, the presence of MVs even at a low rate is a major factor of gene cluster instability. Our study highlights the need for a systematic assessment of imputation methods and so of dedicated benchmarks. A noticeable point is the specific influence of some biological dataset.

## Background

Numerous genomes from species of the three kingdoms are now available [1,2]. A major current aim of biological research is to characterize the function of genes, for instance their cellular regulation pathways and implications in pathology [3-7]. High-throughput analyses (*e.g*., Microarrays) combined with statistical and bioinformatics data analyses are necessary to decipher such complex biological process [8,9]. Microarrays technologies allow the characterization of a whole-genome expression by measuring the relative transcript levels of thousand of genes in one experiment [10,11]. For instance, their relevancies were proved for the classification/identification of cancer subtype or diseases [12-17].

However, technical limitations or hazards (dust, scratches) lead to corrupted spots on microarray [18]. During the image analysis phase, corrupted or suspicious spots are filtered [11], generating missing data [18]. These missing values (MVs) disturb the gene clustering obtained by classical clustering methods, *e.g*., hierarchical clustering [19], *k-means *clustering [20], Kohonen Maps [21,22] or projection methods, *e.g*., Principal Component Analysis [23]. In practice, three different options can be considered. The first method leads to the elimination of genes, *i.e*., information loss [5]. The eliminated genes may be numerous and among them some may be essential for the analysis of the studied mechanism [24]. The second method corresponds to the replacement by zero [13]; it brings up a different problem in the analysis. Indeed, real data close to 0 will be confused with the MVs. Thus to limit skews related to the MVs, several methodologies using the values present in the data file to replace the MVs by estimated values have been developed [25].

The most classical method to estimate these values is the *k*-nearest neighbours approach (*kNN*), which computes the estimated value from the *k *closest expression profiles among the dataset [26]. This approach was applied to DNA chips by Troyanskaya and collaborators [27] and rapidly became one of the most popular methods. Since this pioneer study, more sophisticated approaches have been proposed, like Sequential *kNN *(*SkNN*) [28].

Simple statistical methods have been also proposed as the *Row Mean *[29]/*Row Average *method [28], or approaches based on the Expectation Maximisation algorithm (EM), *e.g*., *EM_gene *and *EM_array *[29]. Principle of least square (LS) has been also widely used, *e.g*., *LSI_gene*, *LSI_array*, *LSI_combined *and *LSI_adaptative *[29]. Kim and co-workers have extended the Least Square Imputation to Local Least Square Imputation (*LLSI*) [28]. However this method is only based on the similarity of genes for estimating the missing data. Others more sophisticated methods like the Bayesian Principal Component Analysis (*BPCA*) [30] combines a principal component regression, a Bayesian estimation and a variational Bayes (VB) algorithm.

The MVs replacement in microarrays data is a recent research field and numerous new and innovative methodologies are developed. We can noticed the work of Bar-Joseph *et al*. who described a model-based spline fitting method for time-series data [31] and Schliep *et al*. who used hidden Markov models for imputation [32]. Tuikkala and co-workers have investigated the interest to use GO annotation to increase the imputation accuracy of missing values [33] as Kim *et al*. [34]. Hu *et al*. and Jörnsten *et al*. have incorporated information from multiple reference microarray dataset to improve the estimation [35,36], while Gan co-workers takes into consideration the biological characteristics of the data [37]. Hua and Lai did not propose a new method, but assess the quality of imputation on the concordance of gene prioritization and estimation of true/false positives [38].

In addition we can list the following relevant methodologies applied in MVs replacement for microarray analysis: *Support Vector Regression *[39], *Factor Analysis Regression *[40], *Ordinary Least Square Impute *[41],* Gaussian Mixture Clustering *[42], *LinCmb *[43], *Collateral Missing Value Estimation *[44], *Linear based model imputation *[45], *Dynamic Time Warping *[46] or *iterative kNN *[47,48].

In a previous study, we estimated the influence of MVs on hierarchical clustering results and evaluated the effectiveness of *kNN *approach [49]. We observed that even a low rate of missing data can have important effects on the clusters obtain by hierarchical clustering methods. Recently, this phenomenon was confirmed by Wong and co-workers for other particular clustering methods [50].

Since our work, numerous replacement methods (see Table 1 and previous paragraphs) have been developed to estimate MVs for microarray data. Most of the time, the new approaches are only compared to *kNN*. In this study, we decided to evaluate the quality of MV imputations with all usable methods, and their influence on the quality of gene clustering. The present paper undertakes a large benchmark of MVs replacement methods to analyze the quality of the MVs evaluation according to experimental type (kinetic or not), percentage of MVs, gene expression levels and data source (*Saccharomyces cerevisiae *and human).

**Table 1:** Different missing values replacement methods.

Methods	Author	Availability	Language	Used	Year
*K*-Nearest Neighbors (*kNN*)	Troyanskaya O.	Y	C	Y	2001
Bayesian Pricipal Component Analysis (*BPCA*)	Oba S.	Y	JAVA	Y	2003
*Row Mean*^1^	Bø T.H.	Y	JAVA	Y	2004
*EM_gene*^1^	Bø T.H.	Y	JAVA	Y	2004
*EM_array*^1^	Bø T.H.	Y	JAVA	Y	2004
*LSI_gene*^1^	Bø T.H.	Y	JAVA	Y	2004
*LSI_array*^1^	Bø T.H.	Y	JAVA	Y	2004
*LSI_combined*^1^	Bø T.H.	Y	JAVA	Y	2004
*LSI_adaptative*^1^	Bø T.H.	Y	JAVA	Y	2004
Sequential KNN (*SkNN*)	Kim K.	Y	R	Y	2004
Local Least Square Impute^2 ^(*LLSI*)	Kim H.	Y	MATLAB	Y	2005
*Row Average*^2^	Kim H.	Y	MATLAB	Y	2005
Linear model based Imputation (*LinImp*)	Scheel I	Y	R	N	2005
FAR, Factor Analysis Regression (*FAR*)	Feten.	N	-	N	2005
Ordinary Least Square Impute (*OLSI*)	Nguyen D.V.	N	-	N	2004
Support Vector Regression (*SVR*)	Wang X.	Y	C++	N	2006
Gaussian Mixture Clustering (*GMC*)	Ouyang M.	On demand	MATLAB	N	2004
Singular Value Decomposition (*SVD*)	Troyanskaya O.	N	C	N	2001
*ghmm*	Schielp, A	Y		N	2003
Collateral Missing Value Estimation (*CMVE*)	Sehgal M.	On demand	MATLAB	N	2005
*GO-based imputation*	Tuikkala	N	-	N	2005
*LinCmb*	Jörnsten, R	On demand	MATLAB	N	2005
Integrative Missing value Estimation *(iMISS)*	Hu, J	Y	C++	N	2006
Projection Onto convex sets (*POCS*)	Gan, X	N	-	N	2006
*Iterative kNN*	Bras	N	-	N	2007

Is given the name of the methods, the authors, its availability, if we have used it (Y) or not (N) and the publication year.

^1 ^*Package *Bø T.H.

^2 ^*Package *Kim H.

## Results

### General principle

**Figure 1:**
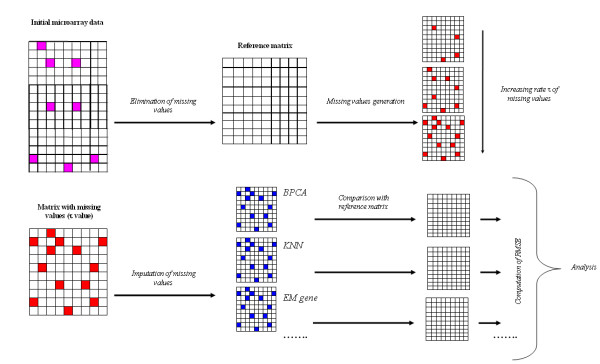
**Principle of the method**. The initial data matrix is analyzed. Each gene associated to at least one missing value (*in pink*) is excluded given a *Reference matrix *without any missing value. Then missing values are simulated (*in red*) with a fixed rate τ. This rate τ goes from 0.5% to 50% of missing values by step of 0.5%. 100 independent simulations are done each time. Missing values are then imputed (*in blue*) for each simulations by the selected methods. *RMSE *is computed between the estimated values of missing values and their true values.

Figure 1 shows the general principle of the analysis. From the initial gene expression datasets, the series of observations with missing values are eliminated to create a *Reference matrix*. Then *simulated *missing values are generated for a fixed τ percentage and are included in the *Reference matrix*. In a second step, these *simulated *missing values are imputed using the different available methods. Difference between the replaced values and the original true values is finally evaluated using the root mean square error (*RMSE*) (see Methods). In this work, we chose 5 microarray datasets, very different one from the other, *i.e*., coming from yeasts and human cells, and with or without kinetics (see Table 2). The idea was to have the broadest possible vision types of expression data [see Additional file 1 for more details [49,51-54]].

**Table 2:** The different datasets used

	Ogawa et *al*., 2000		Gasch et *al*., 2000		Bohen S.P et *al*., 2002	Lelandais *et al*., 2005
**Organism**	***Saccharomyces cerevisiae***		***Saccharomyces cerevisiae***		**human**	***Saccharomyces cerevisiae***

Initial gene number	6013		6153		16523	5261
Initial number of conditions	8		178		16	6
Missing values (%)	0.8		3		7.6	11.4
Genes with missing values (%)	3,8		87,7		63,6	88.29
Genes erased from the study	230		NA		NA	616
Conditions erased from the study	0		136		0	0

	**Ogawa_Complet (OC)**	**Ogawa_subset (OS)**	**Gasch HEAT (GHeat)**	**Gasch H2O2 (GH_2_O_2_)**	**Bohen (B)**	**Lelandais (L)**

Kinetics	N	N	Y	N	N	Y
Final gene numbers	5783	827	523	717	861	4645
Final condition number	8	8	8	10	16	6

Our goals were also (i) to evaluate methods that experimental scientists could use without intervention, (ii) to select only published methods, and (iii) to analyse influence of the gene clusters. Indeed, some studies have been done to compare numerous methods, *e.g*., [55], but does not go through the clustering; while less frequent researches goes through the clustering, but test only a limited number of imputation methods as [56]. We so have searched all kinds of published imputation methods with available dedicated softwares or codes, whenever the Operating System, language or software. From this search, we selected 12 available replacement methods, which were compatible with high-throughput computation. Others methods had not been used due to the unavailability of the program despite the indication in the corresponding papers or to impossibility to modify the source code to used our microarrays data.

### Error rate for each replacement method

**Figure 2:**
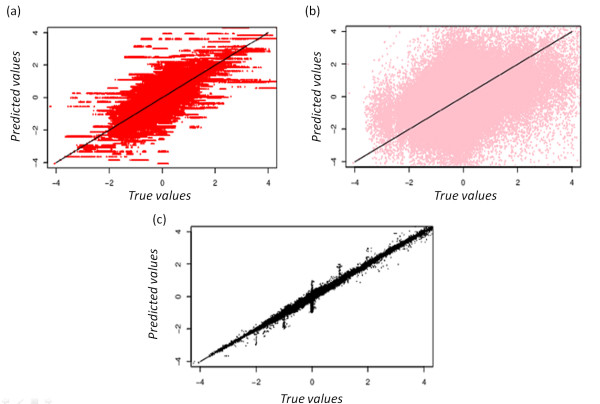
**Example of three methods**. Distribution of predicted values (y-axis) in regards to true values (x-axis). Estimation of the missing values has been done (a) by *kNN *approach, (b) *EM_gene *and (c) *EM_array*. The dataset used is the Bohen set with τ values ranging from 0.5% to 50% of missing values with a step of 0.5. 10 independent simulations have been done for each τ value.

**Figure 3:**
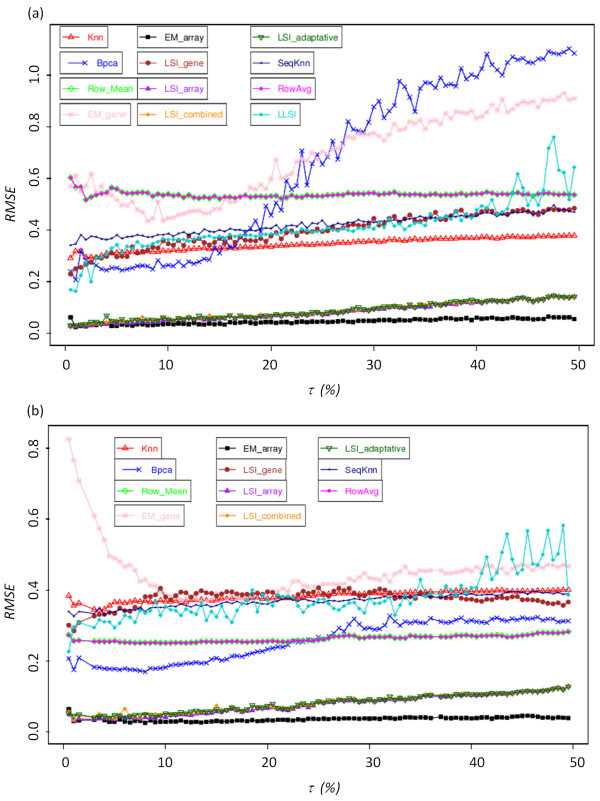
**Missing value imputation**. RMSE value for (a) G_Heat _subset and (b) for OS for rate of missing value going from 0.5% to 50% by step of 0.5%. (b) 100 independent simulations are done at each level.

Figure 2 shows the dispersion of expected and true values, for three given imputation methods. On one hand, *kNN *and *EM_gene *approaches exhibit a high dispersion between expected and true values; the correlations *R *equal respectively 0.33 and 0.32 (see Figures 2a and 2b). On the other hand *EM_array *approach presents a highly better agreement with a *R *value of 0.97 (see Figure 2c). Figure 3 shows the evolution of RMSE values for τ ranging between 0.5 and 50% using the two datasets G_Heat _and OS. These two examples are good illustrations of the different behaviours observed with the different replacement methods. Some have initial high RMSE values and remains quite consistent, while others have lower initial RMSE values but are very sensitive to an increased rate of MVs. Moreover, performances for the different methods appeared to be dependant of the used dataset.

• *EM_gene*[29]: This method is always associated to very high RMSE values, which range in an interval from 0.6 to 0.7 for a rate τ ranging from 0.5 to 3.0% (see Figure 3b) and decrease for values from 0.30 to 0.40. Such a curved profile is observed for the datasets OS and GH_2_O_2 _(see Figure 3a). For the other dataset, RMSE increases as expected (see Figure 3a), but is always associated to high RMSE values.

• *kNN*[27]: Its RMSE values for all six data files always range between 0.3 and 0.4. The increase of τ only affects slightly the *kNN *approximation, at most 0.05 for the datasets B and OS. This constancy of RMSE values implies that for high rates of missing data (more than 20% of missing data) the RMSE values remain acceptable.

• *SkNN*[28]: Despite the fact that *SkNN *is an improvement of *kNN*, their RMSE values are surprisingly always higher than the one of *kNN *(from 0.01 to 0.08). Only with the dataset B, *SkNN *performs slightly better than *kNN *(RMSE difference of 0.076).

• *LLSI*[57]: The average RMSE values of *LLSI *ranges mainly from 0.34 to 0.41 for most of the dataset. Its performance could be considered as median and its effectiveness is close to the *LSI_gene *method. Its RMSE values increase gradually with the increase of τ, *i.e*., 0.1 from 0.5 to 50% of missing data. It is the less efficient method based on least square regressions. However for the dataset L, this method is the most powerful after the *LSIs *methods (see below).

• *LSI_gene*[29]: The effectiveness of *LSI_gene *is slightly affected by the increase in the percentage of missing data. For each data file, the values of RMSE range between 0.3 and 0.4. These results are close to those observed for methods *LLSI *and *kNN*, *i.e*., methods giving of the medium results ranging between the best (*LSI_array*) and the less efficient methods (*EM_gene*).

• *Row Mean*[29] and *Row Average *[57]: Low RMSE values are observed for L (0.23) and B (0.28) datasets. Only for dataset GHeat, the RMSE value is high (0.54). Strikingly this method shows equivalent and or better results than more elaborated approaches.

• *BPCA*[30]: For the OC, OS and GH_2_O_2 _datasets, and for τ comprises in the range 0.5 to 10-15% of missing data, *BPCA *appears to have one of the lowest RMSE values [see Additional file 2], only bypass by two other approaches. This method is powerful for low rates of missing values. However it should be noted that the efficiency of *BPCA *is strongly reduced when the rate of missing data increases. This is particularly notable in the case of the GHeat dataset. The values of the RMSE increases from 0.2 to 1.1 (see Figure 3a). For a τ value higher than 30%, *BPCA *performs worst than most of the imputation methods. This observation is less striking for the other datasets. For B and OS datasets, RMSE values increase by a maximum of 0.1 for τ increasing from 0.5 to 50%. It is a good illustration of the dataset specificity related to the quality of the imputation methods.

• *LSI_array*, *LSI_combined*, *LSI_adaptative *and *EM_array*[29]: Their RMSE values are always lower than 0.1. Remarkably, it is true even for a rate of missing data that equals 50%. The average RMSE values of *EM_array *are slightly lower than the ones of the three other methods. It is striking when the rate of missing data exceeds 20%. A pair-wise comparison shows that *EM_array *is better than the three other methods; its approximation is better in 2/3 of the case. If τ is higher than of 33%, this method remains the best one in 80% of the cases (see Table 3 for two examples).

**Table 3:** Pairwise comparison of imputation method.

(a)										
	***kNN***	***BPCA***	***Row Mean***	***EM_gene***	***EM_array***	***LSI_gene***	***LSI_array***	***LSI_combined***	***LSI_adaptative***	***SkNN***
*kNN*	-----	23.47	47.65	60.82	4.59	38.06	5.00	5.41	7.25	47.14
*BPCA*	-----	-----	75.41	81.33	11.12	67.04	12.76	14.49	16.63	75.51
*Row Mean*	-----	-----	-----	64.69	4.49	40.82	5.10	5.71	6.12	52.45
*EM_gene*	-----	-----	-----	-----	3.67	29.49	4.08	4.39	5.31	37.04
*EM_array*	-----	-----	-----	-----	-----	92.45	**60.04**	**63.89**	**63.36**	95.00
*LSI_gene*	-----	-----	-----	-----	-----	-----	7.86	7.65	7.45	61.53
*LSI_array*	-----	-----	-----	-----	-----	-----	-----	37.24	38.27	94.79
*LSI_combined*	-----	-----	-----	-----	-----	-----	-----	-----	44.39	93.78
*LSI_adaptative*	-----	-----	-----	-----	-----	-----	-----	-----	-----	92.96
*SkNN*	-----	-----	-----	-----	-----	-----	-----	-----	-----	-----
										
(b)										
	***kNN***	***BPCA***	***Row Mean***	***EM_gene***	***EM_array***	***LSI_gene***	***LSI_array***	***LSI_combined***	***LSI_adaptative***	***SkNN***
*kNN*	-----	42.59	44.02	55.90	6.32	45.45	18.74	18.74	18.74	50.09
*BPCA*	-----	-----	52.02	63.49	7.84	53.04	23.37	23.37	23.37	58.03
*Row Mean*	-----	-----	-----	62.18	6.69	24.88	14.01	14.01	14.01	56.58
*EM_gene*	-----	-----	-----	-----	5.06	39.27	15.67	15.67	15.67	44.54
*EM_array*	-----	-----	-----	-----	-----	92.97	**79.65**	**79.65**	**79.65**	93.61
*LSI_gene*	-----	-----	-----	-----	-----	-----	14.85	14.85	14.85	55.52
*LSI_array*	-----	-----	-----	-----	-----	-----	-----	39.24	43.29	81.67
*LSI_combined*	-----	-----	-----	-----	-----	-----	-----	-----	46.49	81.67
*LSI_adaptative*	-----	-----	-----	-----	-----	-----	-----	-----	-----	81.67
*SkNN*	-----	-----	-----	-----	-----	-----	-----	-----	-----	-----

Is given the percentage of better approximation of one method versus another for a rate of missing value t equal to (a) 32% and (b) 48.5% with the OS dataset. The percentage is given in regards to the method given at the left.

### The different datasets influence the quality of the imputation

Table 4 shows the average RMSE values for each imputation methods. They are given as the average of all the simulations ranging from τ = 0.5 to 50% (50,000 independent simulations per imputation method). This table highlights the differences that were observed between the datasets. Nonetheless, it allowed us to rank the methods in term of efficiency. Roughly, we could identify three groups: The first one comprise four methods (*EM_array*, *LSI_array*, *LSI_combined *and *LSI_adaptative*) for which small RMSE values were always observed (*EM_array *always exhibited the best performances); (2) the second group comprised 4 methods, *i.e., BPCA*, *Row Mean*, *LSI_gene *and *LLSI*; (3) and finally the third group, which can be considered as the last group, comprised three methods, *i.e*.,* kNN*, *SkNN *and *EM_gene*.

**Table 4:** Mean RMSE value for the different datasets

		methods									mean
		*EM_gene*	*SkNN*	*kNN*	*LLSI*	*LSI_gene*	*Row Mean*	*BPCA*	*LSI_array*	*EM_array*	
datasets	B	0.334	0.390	0.455	0.344	0.320	0.283	0.194	0.098	0.053	0.275
	GH_2_O_2_	0.586	0.445	0.431	0.452	0.358	0.319	0.334	0.068	0.028	0.336
	OS	0.444	0.369	0.383	0.379	0.377	0.263	0.257	0.077	0.036	0.287
	L	0.388	0.292	0.300	0.078	0.261	0.215	0.250	0.028	0.020	0.204
	GHeat	0.703	0.426	0.350	0.412	0.403	0.541	0.690	0.091	0.054	0.408
	mean	0.491	0.384	0.384	0.333	0.344	0.324	0.345	0.072	0.038	0.302

Notably, this order depends on the dataset, but still the changes are often limited. For instance, *EM_gene *performs better than *kNN *and *SkNN *for B dataset, but does not perform better than the others. Strong changes could be noted for OS that allows *SkNN *to be better than *LLSI *and *LSI*_*gene*. Nonetheless, it is mainly due to the poor quality of the estimation of these two methods with this dataset. For the L dataset, we observed that *LLSI *method performs well and remains better than other *LSIs *and *EM_array *methods. GHeat dataset that is associated to the highest average RMSE values has strong particularities as (i) *kNN *performs better than *BPCA*, *Row Mean*, *LSI_gene *and *LLSI*, and (ii) *BPCA *and *Row Mean *performs poorly compared to other methods, being only slightly better than *EM_gene*. Hence, it appears that GHeat is a more difficult dataset to impute.

### Extreme values

**Figure 4:**
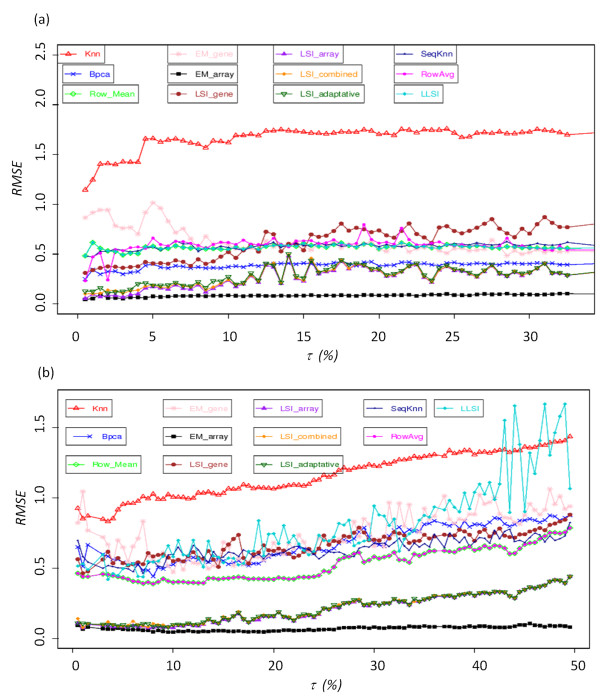
**Extreme values (representing 1% of the missing values)**. Evolution of RMSE according to τ ranging (a) from 0.5% to 30% of the extreme values for the Bohen dataset and (b) from 0.5% to 50% of the extreme values) for the Ogawa dataset.

The same methodology was followed to analyze the extreme values, *i.e*., 1% of the microarray measurements with the highest absolute values. They have major biological key role as they represent the highest variations in regards to the expression reference [see Additional file 3]. Figure 4 presents similar examples to these of Figure 3, but this time, only extreme values were used in the analysis. Thus, the percentage of missing values τ can be differently apprehend, *i.e*., τ = 10% corresponds to 10% of the extreme missing values, so 0.1% of the values of the dataset. At one exception, all the replacement methods decrease in effectiveness for the estimate of the extreme values. Performance of the methods also greatly depends on the used dataset and especially -in agreement with previous observation - in the case of the GHeat dataset. A description of the behaviour of each method is presented in Additional file 3. *kNN *[27] is the less powerful method in most of the case (see Figures 4a and 4b). Its average RMSE value is often 0.5 higher than the second poorest imputation method. Interestingly, in the case of the extreme values, *SkNN *improved greatly. *EM_gene *[29] remains one of the less powerful methods for the imputation of missing values. *LLSI *[57] method effectiveness remains similar compared to the other methods of its group. *Row Mean *[29] and *Row Average *[57] have RMSE values increased by 0.2 to 0.4 for the yeast dataset, which is correct in regards to other methods (see Figures 6). Their efficiencies are median compared to the other methods. *BPCA *[30] has a correct behaviour. But contrary to most of them, it is very sensitive to the datasets. *LSI_gene *[29] has the lowest RMSE values observed after *EM_array*, *LSI_array*, *LSI_combined *and *LSI_adaptative*. This result shows that *LSIs*, whatever the specificity of their implementations, are effective to impute the values missing.

*EM_array *method is again the most performing method (see previous section). Its RMSE values are almost identical to the ones previously computed. *LSI_array*, *LSI_combined *and *LSI_adaptative *are slightly less efficient than previously seen. Thus, the clustering we have proposed remains pertinent when only the extreme values are implicated. *LSI*_*array*, *LSI_combined*, *LSI_adaptative *and *EM_array *are always good, and the less efficient methods can be associated now to considerable RMSE values. Noticeably, *kNN *efficiency collapses and the influence of datasets on the imputation quality is sharpened.

### Clustering in question

A critical point in the analysis of DNA data is the clustering of genes according to their expression values. Missing values have an important influence on the stability of the gene clusters [49,58]. Imputations of missing values have been used both to do hierarchical clustering (with seven different algorithms) and *k-means *[20] (see Methods).

**Figure 5:**
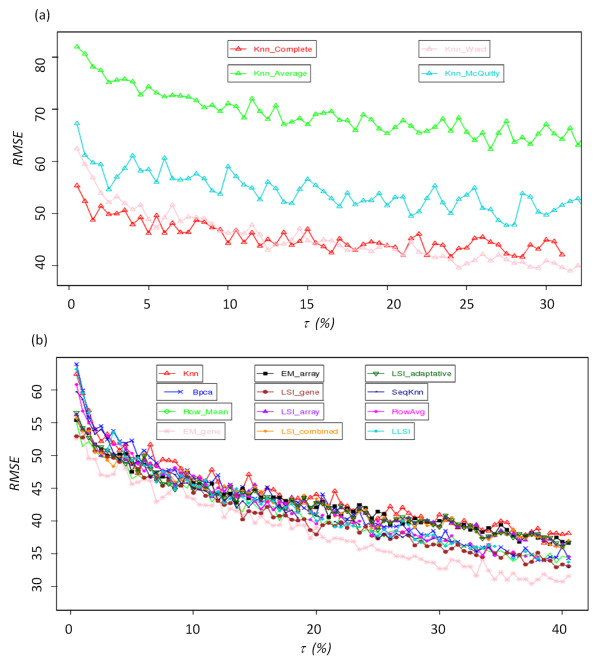
***CPP *of hierarchical clustering approach algorithm**. (a) with complete, average, ward and McQuitty algorithm for OS with *kNN *and (b) with Ward algorithm for Ogawa dataset for the different imputation methods.

Figure 5a shows the Cluster Pair Proportions (*CPP*, [49] see Methods section) of OS using hierarchical clustering with *complete linkage*, *average linkage*, *McQuitty *and *Ward *algorithm. *CPP *values of *average linkage *ranges between 78 and 68%, those of *McQuitty *between 58 and 45%, those of *Ward *between 57 and 35% and finally those of *complete linkage *between 50 and 41%. We obtain for the 7 hierarchical clustering algorithms the same behaviours than previously observed [49]: ranging from high *CPP *values for *single linkage *to low *CPP *values for *Ward*. This observation can be explained by the topology given by each algorithm, *e.g*., *Ward *gives well equilibrated clusters whereas *single linkage *creates few major clusters and numerous adjacent singletons.

For every hierarchical clustering methods the *CPP *values are different, but the general tendencies remain the same: (i) imputation of small rate τ of MVs has always a strong impact on the *CPP *values, and (ii) the *CPP *values slowly decreased with the increased of τ. Between 0.5 and 3% of MVs and the CPP values decrease by 1 to 3% per step of 0.5% of MVs. From τ equals 3.5 to 20% of MVs, the values of *CPP *decrease overall by 10%. For higher rate of MVs the decreasing of CPP is slower. This loss of stability is present in the case of the *k-means *method and for each type of hierarchical classification (except for the methods *single linkage *and *centroid linkage*, due to the building of the clusters).

Individual evaluation of the methods highlights the lack of efficiency of the *EM_gene *imputation method; it obtains always the lowest *CPP *values, *i.e*., 1.37 to 5.34% less than other approaches. At the opposite, *EM_array*, *LSI_array*, *LSI_combined *and *LSI_adaptative *are associated to the highest *CPP *values. In the case of the methods with a median efficiency, *e.g*., *Row_Mean*, their *CPP *values could be assigned as median compared to the values of the other methods. Figure 5b shows the particular example of OS dataset. *CPP *values of BPCA (average value equals 42.6%) are close to the most powerful methods (42.8% for the four methods). Moreover, in the classical range of τ less than 20%, it is the best. As seen in Table 4, BPCA is one of the best approaches for this dataset. Hence, common trends can be found between the quality of the imputation method and the gene cluster stability.

In addition, evaluation of imputation methods shows that the cluster quality depends on the dataset. For instance, with the dataset OS, imputation of missing values with *kNN *method gives an average *CPP *value (for the *Ward *algorithm) that equals 42.9%, while the average *CPP *values for all the other methods only equals 40.6% whereas its RMSE value is one of highest (see Table 4). The *CPP *differences are mainly bellow 5%. These results show that an improvement has been obtained since last study. Nonetheless, no new approaches had drastically improved the quality of the clustering. Interestingly, *k-means *approach had similar tendencies, underlining that this low improvement is not due to hierarchical clustering.

Another question is the comparison between hierarchical clustering algorithms and *k-means*. Nonetheless, comparison only between hierarchical clustering algorithms is already a difficult task. Comparison with *k-means *is so more difficult. Indeed, the use of the same number of clusters to compare the hierarchical clustering algorithms with *k-means *can leads to a wrong conclusion. Indeed, for an equivalent number of clusters, most of the *CPP *values of *k-means *are lower than *CPP *values obtained with hierarchical clustering algorithms. However, it is only due to the dispersion of observations within the clusters obtained by *k-means *approach. Thus, to have an unbiased comparison, the dispersion of genes within cluster between *k-means *and hierarchical clustering algorithms must be computed. It had been done, as previously described [49]. Following this approach, *Ward *and *complete *linkages were defined as the best approaches to assess an unbiased comparison. They have both *CPP *values lower than *k-means CPP *values. The differences were often higher than 5% underlining the interest of *k-means *approach to cluster gene expression profiles.

### Distribution of the observations

When index *CPP *is calculated, only one group is taken into account. To go further, we used another index, named *CPP_f _*that allows to take into account the five closer groups, and to check the pairs of genes remaining joint partners. The values of *CPP_f _*are higher than those of the *CPP*, *e.g*., 20% for the Ward. Methods associated to high *CPP *values have also high *CPP_f _*values, while methods with low *CPP *values have also a lower *CPP_f _*values. These weak variations shows that often a part of the observations, not associated to the original cluster could be find in its vicinity. These results are entirely in agreement with our previous results [49]. It shows here that the novel imputation methods have not permit to get closer related genes with better improvement.

The analysis of associations could also take into account the non-associations. For this purpose, Clustering Agreement Ratio (*CAR*, see Methods section) has been used which considers both associated and non-associated genes. *CAR *values are higher than the one of the *CPP *due to the calculation of the pairs of genes remaining dissociated. Indeed, it is more probable than the genes are dissociated than associated according to the number of treated genes and the number of generated groups. For the OS dataset, the highest values of the index CAR concerns *Ward *classification and are ranging between 88.2 and 91.2%. For the GHeat dataset, it ranges between 91.0 and 94.1%. *Complete linkage*, *average linkage *and *McQuitty *have lower CAR values (80%). For *k-means *classification, the values are higher 1 to 2% compared to *Ward *classification, 10% better than *McQuitty *and *Complete linkage *and 13% to *average linkage*. This results underlines that *K-means *allows so a better stability of gene clusters.

## Discussion

### Imputation

Since our previous analysis [49], numerous new MVs imputation methods have been proposed. Some appeared to be true improvements in regards to the computation of RMSE. In particular, *EM_array *is clearly the most efficient methods we tested. For τ < 35%, it is the best imputation method for 60% of the values, and for τ > 35%, in 80%. This feature was confirmed by the analysis of extreme values. *LSI_array*, *LSI_combined *and *LSI_adaptative *follow closely the efficiency of *EM_array*. We have unsuccessfully tried to combine these four different methods to improve the RMSE values. No combination performs better than *EM_array*.

We can underline four interesting points:

i. As expected, the imputation quality is greatly affected by the rate of missing data, but surprisingly it is also related to the kind of data. *BPCA *is a perfect illustration. For non-kinetic human dataset, MVs estimations were correct, whereas for the GHeat dataset the error rate appeared to be more important.

ii. The efficiency of *Row_Mean *(and *Row_Average*) is surprisingly good in regards to the simplicity of the methodology used (with the exception of GHeat dataset).

iii. Even if *kNN *is the most popular imputation method; it is one of the less efficient, compared to other methods tested in this study. It is particularly striking when analyzing the extreme values. *SkNN *is an improvement of *kNN *method, but we observed that RMSE values of *SkNN *were not better than ones of *kNN*. It could be due to the use of non-optimal number of neighbours (*k*), as for *kNN*. It must be noticed we used *k*_opt _defined by [27], this choice has a direct impact on the imputation values.

Extreme values are the ones that are the most valuable for the experiments. The imputation of extreme value missing data shows that -except for *EM_array*- the effectiveness of all the methods is affected.

Our results are so in good accordance with the results of Brock and co-workers [55] who found that methods from Bo and co-workers [29], Kim and co-workers [57] and Oba and co-workers [30] are highly competitive. However, they consider "that no method is uniformly superior in all datasets" [29]. Our results are simpler to summarize as we observe -thanks to our distance criteria- a grading between the effectiveness of the methods. *LLSI *of Kim and co-workers [57] has a correct behavior for all datasets while *BPCA *of Oba and co-workers [30] is strongly dependant of the dataset. At the opposite, the methods implemented by Bo and co-workers [29] remain the most efficient in all cases. Moreover, some implemented methods of Bo and co-workers [29] have not been tested by [55], but are the most efficient. All these results are reinforced by the analyses of extreme value imputations.

An important point must be not forgotten, we have, as the other authors, *e.g*., [24,55,56], used the entire dataset, *i.e*., no specific selection of interesting profile gene had been done. It could have importance in terms of quality of the imputation values and consequence on the clustering.

### Clustering

A strong assumption of the microarray data analysis is that genes with similar expression profiles are likely to be co-regulated and thus involved in the same or similar biological processes. Different types of clustering and classification methods have been applied to microarray data, *e*. *g*., some classical as *k-means *clustering [20], self-organizing maps [21,22,59], hierarchical clustering [19,60], Self Organizing Tree Algorithm [61-63], and some dedicated approaches as DSF_Clust [64], re-sampling based tight clustering [65], cluster affinity search technique [66], multivariate Gaussian mixtures [67], model-based clustering algorithms [68,69], clustering of change patterns using Fourier coefficients [70], Nearest Neighbor Networks [71], Fuzzy clustering by local Approximation of membership [72] or Multi-Dimensional Scaling [73].

Given one particular dataset, different clustering algorithms are very likely to generate different clusters [74]. This is true when large-scale gene expression data from microarrays are analyzed [58,75,76]. Comparison of different clusters even obtained with the same classification approach is still a difficult task [see Additional file 4[69,77-79]]. Thus, to assess the relevance of missing value imputation methods, we observed the behaviours of different hierarchical clustering methods and *k-means *clustering using *CPP*, *CPP*_f _[49] and newly introduce *CAR *index. Results follow exactly the observations done on RMSE values (see previous section). Only one method seems ambiguous: *kNN*. Indeed, its *CPP *and *CPP*_f _are higher than expected. It is mainly due to the selection of the genes in the different datasets. We have decided at the beginning to not discard any genes, *i*. *e*., we have absolute no *a priori*. Thus very flat profiles have been conserved and empower *kNN *that prefers to predict values closer to zero than the other methods (see Figure 4 of [49]). It generates clusters with lot of zero, these clusters are so stable. For the majority of the methods, the order of effectiveness of the methods for the maintenance of stability within the groups between various classifications is identical. Combination of *CPP*, *CPP*_f _and *CAR *index underlines the interest of *k-means *clustering in regards to hierarchical clustering methods. For comparable clusters, *k-means *gives better values.

Wang and co-workers does not found a strong difference between the three imputation methods they used, *i.e., kNN*, *BPCA *and *LLS*, in the classification performance [24]. The only comparable extensive study has been done by Tuikkala and co-workers [56], they have focussed interestingly on the GO term class and use *k-means*. They have tested six different methods with less simulation per missing value rates and less missing value rates. But, the important point is they have not tested the methods found the most efficient by our approach. We also slightly disagree with their conclusion about the quality of BPCA [56]. It can be easily understand as only a very limited number of clusters has been tested (5 clusters); in our case, we have supervised the choice of cluster numbers (see Method section), leading to a higher number of clusters. This higher number is so more sensitive to the quality of clustering. It must be noticed we have used Euclidean distance and not Pearson correlation, it was mainly to (i) stay consistent with our previous research, and (ii) as we have not filtered the data, Pearson correlation could have aggregated very different profiles. As the time computation was very important, it was not possible to test the two possibilities.

## Conclusions

**Figure 6:**
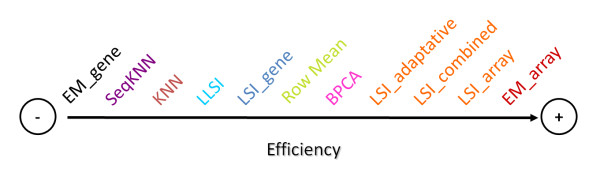
**Summary of the comparison**.

The DNA microarrays generate high volume of data. However they have some technical skews. Microarrays studies must take into account the important problem of missing values for the validity of biological results. Numerous methods exist to replace them, but no systematic and drastic comparisons have been performed before our present work. In this study, we have done more than 6.000.000 independent simulations, to assess the quality of these imputation methods. Figure 6 summarizes the results of our assessment. The method *EM_array*, *LSI_array*, *LSI_combined *and *LSI_adaptative *are the most performing methods. *BPCA *is very effective when the rate of missing values is lower than 15%, *i.e*., for classical experiments. The values estimated by the *Row_Mean *are quite correct in regards to the simplicity of the approach. *kNN *(and *SkNN*) does not give impressive results, it is an important conclusion for a method used by numerous scientists. The methods *LSI_gene *and *EM_gene *are not effective but they are to be tested with data files made up of little of genes and a great number of experiments. These conclusions are to be taken carefully because the quality of the imputations depends on the used datasets.

A major disadvantage of numerous methods is their accessibilities. We have tested here only a part of the methods as some are unavailable and others had not worked properly. Some methods used here could not be used easily by a non-specialist. It could be interesting so to have implementation of all the different methods in a useful manner with the standardized input and output file format. In the second time, graphic interfaces for the methods could be helpful. These remarks are particularly relevant in regards to recent papers that proposed novel approaches as SLLSimpute [80] or interesting comparison [55,56] that do not compare with the methods that had been considered as the most efficient in this study.

## Methods

### Datasets

We used 5 data sets for the analysis [see Additional file 1]; they were mainly coming from the SMD database [81]. The first one, named Ogawa set, was initially composed of *N *= 6013 genes and *n *= 8 experimental conditions about the phosphate accumulation and the polyphosphate metabolism of the yeast *Saccharomyces cerevisiae *[51]. The second one corresponds to various environmental stress responses in *S. cerevisiae *[52]. This set, named Gasch set, contains *N *= 6153 genes and *n *= 178 experimental conditions. Due to the diversity of conditions in this set, we focused on two experimental subsets corresponding to heat shock and H_2_O_2 _osmotic shock respectively. Bohen and co-workers have analyzed the patterns of gene expression in human follicular lymphomas and the interest of treatment by rituximab [53]. This dataset is composed of *N *= 16.523 genes and *n *= 16 experimental conditions. The last dataset has been obtained by Lucau-Danila, Lelandais and co-workers [54]. To precisely describe the very early genomic response developed by yeast to accommodate a chemical stress, they performed a time course analyses of the yeast gene expression which follows the addition of the antimitotic drug benomyl. The dataset is a kinetic that comprised *N *= 5.621 genes for *n *= 6 kinetic time (30 seconds, 2, 4, 10, 20 and 40 minutes).

### Datasets refinement: missing values enumeration

From the original datasets, we built complete datasets without MVs. All the genes containing at least one missing value were eliminated from the Ogawa set (noted OS). The resulting OS set contains *N *= 5783 genes and *n *= 8 experimental conditions. The second set without MVs was taken from Gasch et al. and called GS. The experimental conditions (column) containing more than 80 MVs were removed. The resulting GS matrix contains *N *= 5843 genes and n = 42 experimental conditions. Two subsets were generated from GS and have been noted GHeat and GH_2_O_2_. They correspond to specific stress conditions as described previously. GHeat and GH_2_O_2 _contain respectively *N *= 3643 genes with *n *= 8 experimental conditions and *N *= 5007 genes with n = 10 experimental conditions.

To test the influence of the matrix size, *i.e*., the number of genes, we built smaller sets corresponding to 1/7 of OS, GS, GHeat and GH_2_O_2_. Principles are described in [49]. For the dataset of Bohen et *al*. (noted B), we have done the same protocol and used a subset representing 1/7 of B, *i.e*., N = 861 genes. For the dataset of Lucau-Danila *et al.: *[54], 11.4% of the genes have at least one missing values. The dataset with no missing values (noted L) was so composed of *N *= 4645 genes.

### Missing values generation

From the sets without MVs, we introduced a rate τ of genes containing MVs (τ = 1 to 50.0%), these MVs are randomly drawn. Each random simulation is generated at least 100 times per experiment to ensure a correct sampling. It must be notices that contrary to our previous work, each gene could contain more than one MV [49].

### Replacement methods

The different packages have been downloaded from the authors' websites (see Table 1). *kNN *has been computed using the well-known *KNNimpute *developed by Troyanskaya and co-workers [27]. The determined *k*_opt _value is associated with a minimal global error rate as defined by Troyanskaya and co-workers [27]. *BPCA *was used without its graphical interface [30] as for the Bo et al. package (Java) [29]. For *LLSI *and *Row_Average*, we have modified the original Matlab code to use our own microarray datasets [57]. *SkNN *was performed with R software [28].

### Hierarchical Clustering

The hierarchical clustering (HC) algorithm allows the construction of a dendogram of nested clusters based on proximity information [19]. The HC have been performed using the "hclust" package in R software [82]. Seven hierarchical clustering algorithms have been tested: *average linkage*, *complete linkage*, *median linkage*, *McQuitty*, *centroid linkage*, *single linkage *and *Ward minimum variance *[83].

The distance matrix between all the vectors (*i.e*., genes) is calculated by using an external module written in C language. We used the normalized Euclidean distance d* to take account of the MVs:(1)

*v *and *w *are two distinct vectors and m is the number of MVs between the two vectors. Thus, (*v*_i _- *w*_i_) is not computed if *v*_i _and/or *w*_i _is a missing value

### An index for clustering results comparison: Conserved Pairs Proportion (*CPP*)

To assess the influence of missing data rates and different replacement methods into clustering results (see Figure 1), we have analysed the co-associated genes of an original dataset (without MVs) compared to these genes location in a set with MVs. A similar approach has been used by Meunier *et al*. on proteomic data [84].

Hence, we realized in a first step the clustering with the data sets without MV by each aggregative clustering algorithm. The results obtained by these first analyses are denoted reference clustering (*RC*). In a second step, we generated MVs in data. The MVs are replaced by using the different replacement methods. Then we performed the hierarchical clustering for each new set. The results obtained by these second analyses are denoted generated clustering (*GC*).We compared the resulting clusters defined in *RC *and *GC *and assessed the divergence by using an index named Conserved Pair Proportions (*CPP*). The *CPP *is the maximal proportion of genes belonging to two clusters, one from the RC and the other one from the GC (cf. Figure 1 of [49] and Additional file 5 for more details).

### Clustering Agreement Ratio (CAR)

The Clustering Agreement Ration (CAR) is the concordance index measuring the proportion of genes pairs, either belonging to a same cluster (resp. different clusters) in the reference clustering (RC) and found again in a same cluster (resp. different clusters) in the clustering (GC) obtained without or after replacing the MVs.

The index CAR is defined by the following equation:(2)

where  and  specify the co-presence of two genes in a same cluster, i.e., they take the value 1 when the genes *i *and *j *belong to a same cluster in the clustering *RC *and *GC *respectively. The numbers of pairs in *G *genes is *G*.(*G *- 1)/2. The first term of the numerator corresponds to the co-presence of the pair (*i*, *j*) in a same cluster for *RC *and *GC*, and, the second term the co-absence of this pair in a same cluster.

## Competing interests

The authors declare that they have no competing interests.

## Authors' contributions

MC done all the computational and analysis works. AdB wrote the paper, conceived of the study and carried out the MVs generation. AM and GL participated in the design of the study and coordination. All authors read and approved the final manuscript.

## Supplementary Material

Additional file 1 **Dataset details**. Click here for file

Additional file 2 **RMSE of OS with BPCA imputing method**. RMSE value for OS for rate of missing value going from 0.5% to 20% by step of 0.5% with the L dataset. Click here for file

Additional file 3 **Extreme values**. Distribution of the values observed in OS dataset. The extreme values are highlighted on each size of the histogram. Click here for file

Additional file 4 **Comparing clustering algorithms**. Click here for file

Additional file 5 **Details of *CPP *and *CPPf***. Click here for file
