# Supplementary material for: Comparative analysis of missing value imputation methods to improve clustering and interpretation of microarray experiments
Source: BMC Genomics. 2010 Jan 7;11:15. doi: 10.1186/1471-2164-11-15 (PMC2827407; doi:10.1186/1471-2164-11-15)
Supplement: Additional file 1 — Dataset details. [file 1471-2164-11-15-S1.DOC]

## Additional file 1 – Dataset details

The data file of Ogawa and collaborators (namely OC dataset) comes from experiments on the yeast *Saccharomyces cerevisiae*. Their work concerned the identification of the components of the system of regulation PHO involved in the acquisition of phosphate [1]. The initial data file comprised expression measurements for 6013 genes in 8 different experimental conditions (several mutant stocks compared with the wild stock with various phosphate concentrations). In a preliminary step, all the pre-existing missing values were eliminated. Only 230 genes were thus removed (3.8%) preserving 5783 genes in the final dataset. We next selected a subset of 827 genes that represented 1/7^th^ of the whole dataset [2]. This subset (namely OS) has the interest to be representative of the different behaviors of the complete dataset, but required less computational time for the replacement of missing values and clustering procedures. The data of Gasch and collaborators also relates to the yeast *Saccharomyces cerevisiae* [3]. It includes 6,153 genes for 178 experiments. As the number of missing values was high; we eliminated all the experimental conditions with more than 80 missing data. It remained 42 conditions. Again, different data files were generated. The first included 5,007 genes and the 10 experiments related to the osmotic shock response of mutant and wild type cell exposed to the H_2_O_2_. Only 1/7^th^ of the genes were finally used in the analysis (717 genes with 10 conditions, dataset GH_2_O_2_). The second comprised 3,643 genes and 8 experiments related to the thermal shock response (at 37°C) for 4 successive times (5, 15, 30 and 60 minutes). With this dataset the influence of the missing data on kinetic experiments could be estimated. A subset of genes was selected (1/7^th^ of the dataset) and corresponded to 523 genes and 8 experiments, namely GHeat dataset. Bohen and collaborators have works on human follicular lymphomas, the goal being to study the effect of the *ritumibax* on patients [4]. This matrix contained 16,523 genes with 16 experiments where the treatments of the patients were different. In the analysis, we used a subset corresponding to 1/7^th^ of the data, *i*.*e*. 861 genes, namely B dataset. A final set came from an experiment carried out by Lucau-Danila and collaborators. The study is related to the determination of the effect of Benomyl stress-induced on *Saccharomyces cerevisiae* cells, according to time [5]. The expression matrix contains 5261 genes and six points of kinetics (30 seconds, 2, 4, 10, 20 and 40 minutes), namely L dataset. The elimination of genes with missing values (11.4 %) reduced the dataset to 4645 genes and 6 experiments.

1. Ogawa N, DeRisi J, Brown PO: **New components of a system for phosphate accumulation and polyphosphate metabolism in Saccharomyces cerevisiae revealed by genomic expression analysis**. *Mol Biol Cell* 2000, **11**(12):4309-4321.

2. de Brevern AG, Hazout S, Malpertuy A: **Influence of microarrays experiments missing values on the stability of gene groups by hierarchical clustering**. *BMC Bioinformatics* 2004, **5**:114.

3. Gasch AP, Spellman PT, Kao CM, Carmel-Harel O, Eisen MB, Storz G, Botstein D, Brown PO: **Genomic expression programs in the response of yeast cells to environmental changes**. *Mol Biol Cell* 2000, **11**(12):4241-4257.

4. Bohen SP, Troyanskaya OG, Alter O, Warnke R, Botstein D, Brown PO, Levy R: **Variation in gene expression patterns in follicular lymphoma and the response to rituximab**. *Proc Natl Acad Sci U S A* 2003, **100**(4):1926-1930.

5. Lucau-Danila A, Lelandais G, Kozovska Z, Tanty V, Delaveau T, Devaux F, Jacq C: **Early expression of yeast genes affected by chemical stress**. *Mol Cell Biol* 2005, **25**(5):1860-1868.
