# Supplementary material for: Comparative analysis of missing value imputation methods to improve clustering and interpretation of microarray experiments
Source: BMC Genomics. 2010 Jan 7;11:15. doi: 10.1186/1471-2164-11-15 (PMC2827407; doi:10.1186/1471-2164-11-15)
Supplement: Additional file 2 — RMSE of OS with BPCA imputing method. RMSE value for OS for rate of missing value going from 0.5% to 20% by step of 0.5% with the L dataset. [file 1471-2164-11-15-S2.DOC]

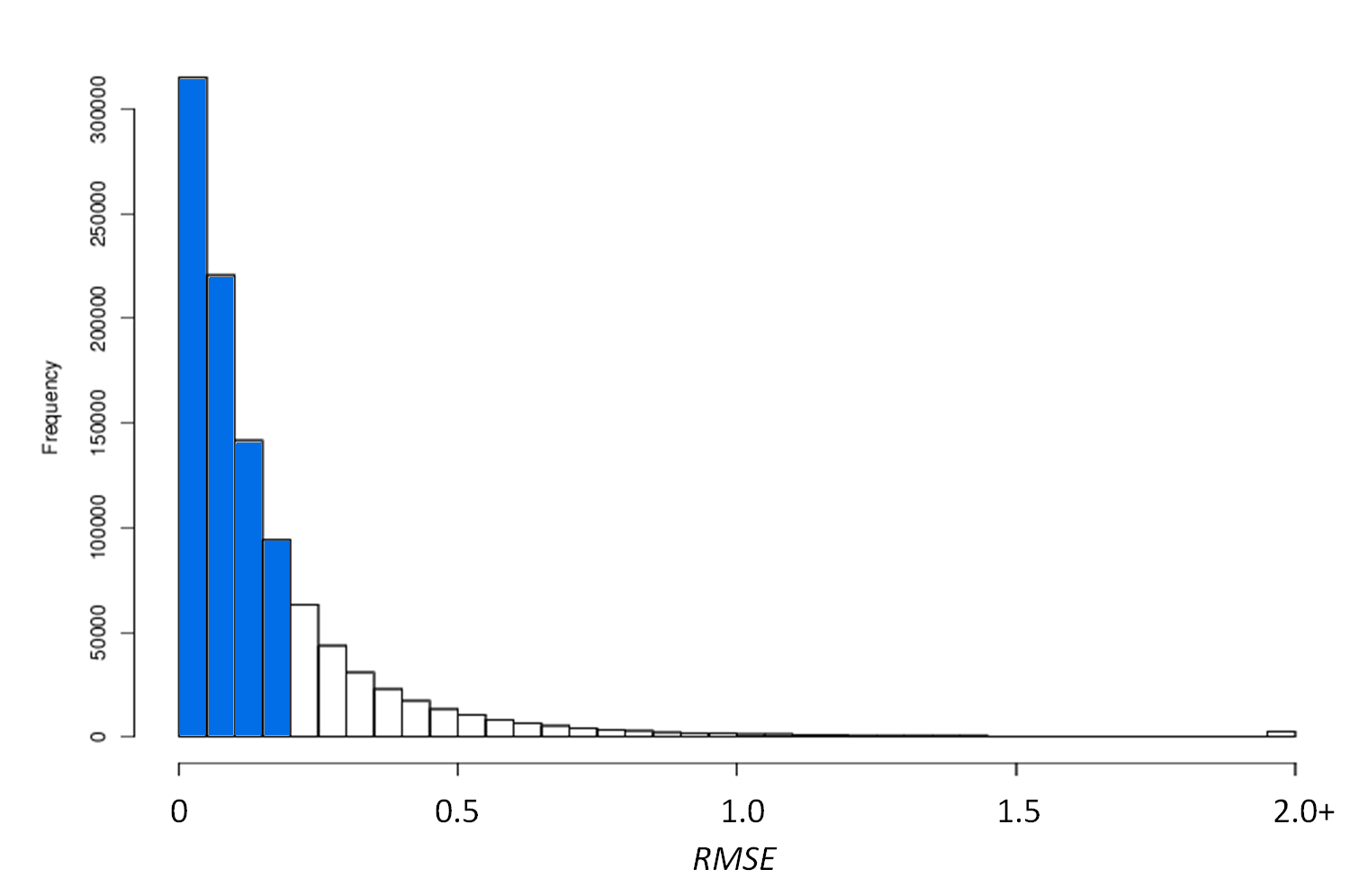


## Additional file 2 – RMSE of OS with BPCA imputing method

RMSE value for OS for rate of missing value going from 0.5% to 20% by step of 0.5% with the L dataset.

This method is powerful for low rates of missing values. As seen here in blue, 76.2% of the data have RMSE values below 0.20. However it should be noted that the efficiency of *BPCA* is strongly reduced when the rate of missing data increases
