# Supplementary material for: Comparative analysis of missing value imputation methods to improve clustering and interpretation of microarray experiments
Source: BMC Genomics. 2010 Jan 7;11:15. doi: 10.1186/1471-2164-11-15 (PMC2827407; doi:10.1186/1471-2164-11-15)
Supplement: Additional file 3 — Extreme values. Distribution of the values observed in OS dataset. The extreme values are highlighted on each size of the histogram. [file 1471-2164-11-15-S3.DOC]

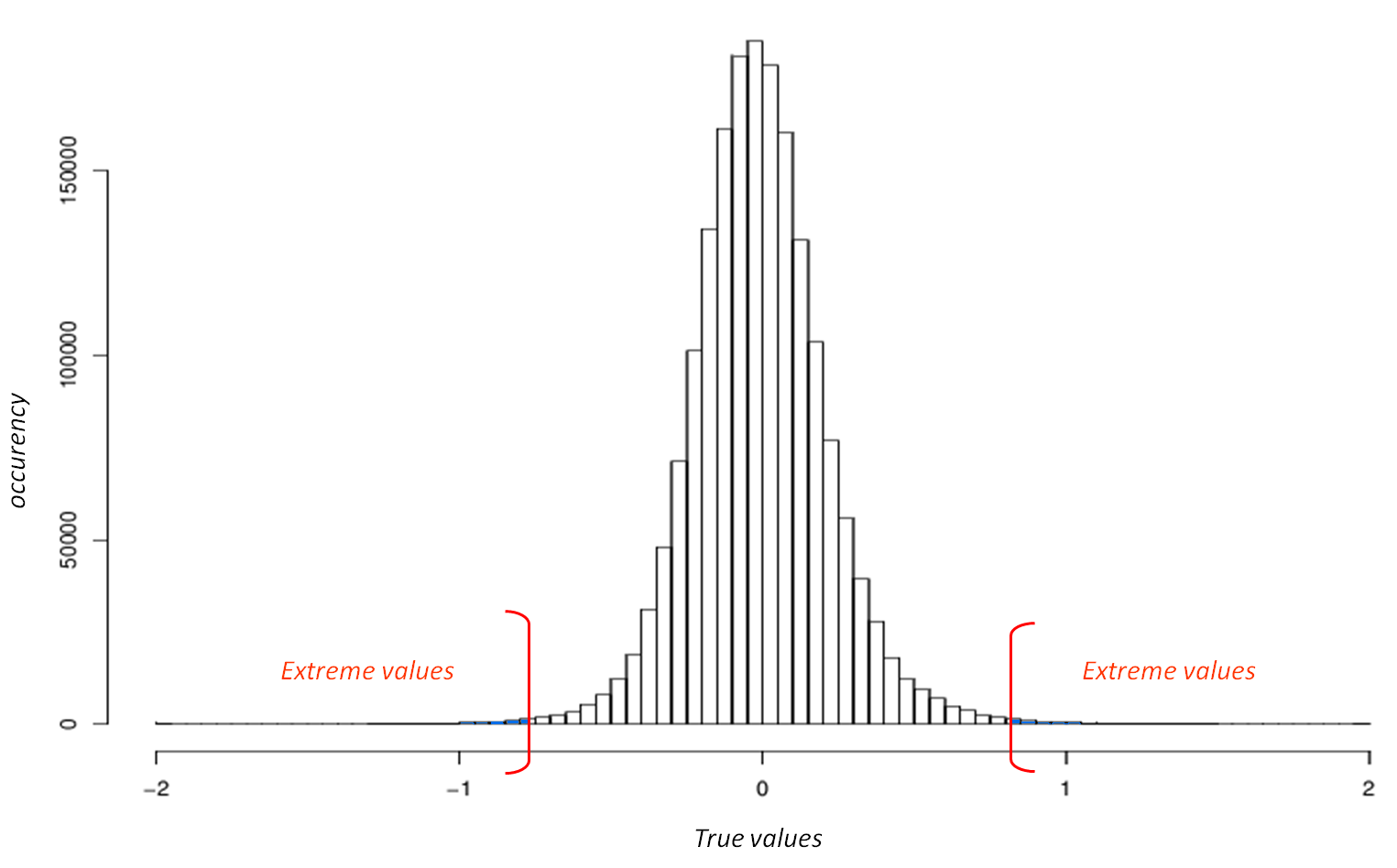


## Additional file 3 – Extreme values

Distribution of the values observed in OS dataset. The extreme values are highlighted on each size of the histogram.

- *kNN* [1]: It is the less powerful method (except for the GHeat dataset). This is well illustrated on Figures 4a and 4b. For instance, its average RMSE value of B dataset increases from 0.45 to 1.66, and its average RMSE value is often 0.5 higher than the second poorest imputation method.
- *SkNN* [2]: Results of *SkNN* were considered comparable to those of *kNN*. But interestingly, in the case of the extreme values, *SkNN* improved greatly. For example with B and OS datasets (see Figures 4a and 4b), RMSE values of *SkNN* is 1.0 and 0.5 below the ones of *kNN*. Similar observations were done for the other datasets.
- *EM_gene* [3]: It remains one of the less powerful methods for the imputation of missing values, but still it is better than *kNN*.
- *LLSI* [4]: It is a method whose effectiveness is similar compared to the other methods. However for the dataset L, the average of the values of the RMSE of method *LLSI* is one of best.
- *Row Mean* [3] and *Row Average* [4]: They have RMSE values increased by 0.2 to 0.4 for the yeast dataset, which is correct in regards to other methods (see Figures 6). Their efficiencies are median compared to the other methods. As for the previous conclusion on *Row Mean*, it is surprising as this method does not use any model of probability and is relatively effective. For GHeat, it is the least performing method when τ ranges between 0.5 and 20 %.
- *BPCA* [5]: From a global point of view, *BPCA* method has a correct behavior. But contrary to most of them, it is very sensitive to the datasets. For instance with Bohen dataset, corresponding to the human cells, its RMSE values are weak remains quite constant (= 0.40). On the other hand and as already observed, for the dataset GHeat, *BPCA* method is very sensitive to the increase in the rate τ. For τ within the range [0.5 to 15%], RMSE values are 0.25 higher than worst methods, but remains low value. For τ more than 15 % of missing data, RMSE values strongly increases and reach an excessive error rate of 3.80. It becomes, consequently, the less efficient method. For the different yeast sets, *BPCA* is misadvised starting from 15 % of missing data.
- *LSI_gene* [3]: It averages RMSE values of *LSI_gene* ranges between 0.6 and 0.8, *i*.*e*. median compared to the other methods. However in the case of the G_Heat_ and G_H2O2_ datasets, from τ equals 0.5 to 20 %, its RMSE values are the lowest observed after *EM_array*, *LSI_array*, *LSI_combined* and *LSI_adaptative*. This result shows that *LSIs*, whatever the specificity of their implementations, are effective to impute the values missing.
- *LSI_array*, *LSI_combined*, *LSI_adaptative* and *EM_array* [3]: *EM_array* method is again the most performing method (see section *Error rate for each replacement method*). Its RMSE values are almost identical to the ones previously computed. Between the average and extreme values only a difference of 0.03 is observed. For *LSI_array*, *LSI_combined* and *LSI_adaptative*, the imputation of extreme values gives also a low error rate. Nonetheless, these three methods are slightly less efficient than previously seen; they had a RMSE value increase of 0.20. However, these four methods remain most effective.

Thus, the clustering we have proposed remains pertinent when only the extreme values are implicated. *LSI*_*array*, *LSI_combined*, *LSI_adaptative* and *EM_array* are always good, and the less efficient methods can be associated now to considerable RMSE values. Noticeably, *kNN* efficiency collapses and the influence of datasets on the imputation quality is sharpened.

1. Troyanskaya O, Cantor M, Sherlock G, Brown P, Hastie T, Tibshirani R, Botstein D, Altman RB: **Missing value estimation methods for DNA microarrays**. *Bioinformatics* 2001, **17**(6):520-525.

2. Kim KY, Kim BJ, Yi GS: **Reuse of imputed data in microarray analysis increases imputation efficiency**. *BMC Bioinformatics* 2004, **5**:160.

3. Bo TH, Dysvik B, Jonassen I: **LSimpute: accurate estimation of missing values in microarray data with least squares methods**. *Nucleic Acids Res* 2004, **32**(3):e34.

4. Kim H, Golub GH, Park H: **Missing value estimation for DNA microarray gene expression data: local least squares imputation**. *Bioinformatics* 2005, **21**(2):187-198.

5. Oba S, Sato MA, Takemasa I, Monden M, Matsubara K, Ishii S: **A Bayesian missing value estimation method for gene expression profile data**. *Bioinformatics* 2003, **19**(16):2088-2096.
