# Supplementary material for: Comparative analysis of missing value imputation methods to improve clustering and interpretation of microarray experiments
Source: BMC Genomics. 2010 Jan 7;11:15. doi: 10.1186/1471-2164-11-15 (PMC2827407; doi:10.1186/1471-2164-11-15)
Supplement: Additional file 4 — Comparing clustering algorithms. [file 1471-2164-11-15-S4.DOC]

## Additional file 4 – Comparing clustering algorithms

Yeung and co-workers have developed a figure-of-merit (FOM) scale [1]. They rated the predictive power of a clustering arrangement based on a leave-one-out technique. This method also tends to diminish the bias of the disequilibrium between the clusters. Wu and co-workers assigned cellular functions with confidence values to new proteins by making use of a database of clusters produced from different clustering algorithms [2]. Thalamuthu and co-workers performed a comprehensive comparative study to evaluate the effectiveness of several commonly used clustering methods [3]. They proposed a weighted Rand index to compare clustering algorithms. Nonetheless, they assessed the performance of the methods only by a predictive accuracy analysis through verified gene annotations. Swift and co-workers improved Wu’s [2] approach to build consensus clusters of gene expression data [4]. This method produces robust clusters which include all full agreement pairs across all clustering methods. As their approach is dependent on some thresholds, it is not obvious that consensus clusters are significantly better than the other clusters.

1. Yeung KY, Haynor DR, Ruzzo WL: **Validating clustering for gene expression data**. *Bioinformatics* 2001, **17**(4):309-318.

2. Wu LF, Hughes TR, Davierwala AP, Robinson MD, Stoughton R, Altschuler SJ: **Large-scale prediction of Saccharomyces cerevisiae gene function using overlapping transcriptional clusters**. *Nat Genet* 2002, **31**(3):255-265.

3. Thalamuthu A, Mukhopadhyay I, Zheng X, Tseng GC: **Evaluation and comparison of gene clustering methods in microarray analysis**. *Bioinformatics* 2006, **22**(19):2405-2412.

4. Swift S, Tucker A, Vinciotti V, Martin N, Orengo C, Liu X, Kellam P: **Consensus clustering and functional interpretation of gene-expression data**. *Genome Biol* 2004, **5**(11):R94.
