# Supplementary material for: Comparative analysis of missing value imputation methods to improve clustering and interpretation of microarray experiments
Source: BMC Genomics. 2010 Jan 7;11:15. doi: 10.1186/1471-2164-11-15 (PMC2827407; doi:10.1186/1471-2164-11-15)
Supplement: Additional file 5 — Details of CPP and CPPf. [file 1471-2164-11-15-S5.DOC]

## Additional file 5 – Details of *CPP* and *CPPf*

The procedure for computing the index *CPP* is as follows:

i) For each reference clustering based on a given clustering algorithm (*algo*), we defined *K*_algo_, the number of clusters. As every type of hierarchical clustering algorithm gives a particular topology, we cannot use the same number of clusters to compare each aggregative method. So, we defined *K*_algo_ such as its 10 most important cluster must represent 80% of the genes. For this purpose, we defined *K*_init_, an important initial number of clusters (equals to 500), and counted the number of occurrences associated to the 10 most populated clusters. Then we diminished *K*_init_ by one unit and counted again. We stopped the process when the 10 most important clusters represent 80% of the occurrences (*K*_algo_ = *K*_init_). We denote by $C_{j}^{ref}$ the jth cluster for a given clustering algorithm with j = {1, … , *K*_algo_}. The $C_{j}^{ref}$ clusters are associated with their corresponding gene list$L_{j}^{ref}$.

ii) Three hierarchical clustering are performed after generating MVs in proportion τ in the data, the first one without replacing data - in this case, the normalized Euclidean distance (Eq.2) is used -, the second one after estimating the missing data by the kNN method (Eq.1), and the third one after replacing the missing data by zero. For each resulting tree, *K*_algo_ clusters are defined. The $C_{j'}^{new}$clusters are associated with their corresponding gene list $L_{j'}^{new}$, with j’ = {1, …, *K*_algo_}.

iii) Finally, to estimate the *CPP* index, we searched for each $C_{j}^{ref}$cluster the closest $C_{j'}^{new}$cluster. For each clustering algorithm (*algo*), the corresponding $C_{j}^{ref}$cluster is selected as the maximum number of genes $G_{j}^{a\lg o}$ from the gene list $L_{j}^{ref}$ found in$L_{j'}^{new}$. Then, the Conserved Pairs Proportion (*CPP*) is computed as follow for one simulation:

$CPP^{a\lg o} =\frac{\sum_{j=1}^{j=K^{\text{algo}}} G_{j}^{a\lg o} }{G}$ (3)

where . The term $\delta_{ii'}$is the Kronecker symbol, *i*.*e*. it is equal to 1 when the genes i and i’ in the two gene lists are identical, otherwise 0. G denotes the total number of genes. This index takes the maximal value 1 when the clusterings RC and GR are identical.

In addition, a variation of the tree topology may induce a *CPP*-variation. If the remaining genes of a cluster are in the direct neighbour clusters, the use of *CPP* can bias the analysis. Thus, we characterized the *CPPf* ratio to consider the f closest clusters of the $C_{j'}^{new}$ cluster. The computation of *CPPf* ratio is based on the previous ratio and corresponds to the f clusters which are the closest to the winning cluster. From a selected$C_{j'}^{new}$, the upper node of the dendrogram is examined. If the number of clusters linked to this node is inferior to *f*, the upper node is selected. This process is performed until the number of clusters is inferior or equals to *f*. The last node tested (*i.e.* with the number of clusters inferior or equal to *f*) is used to compute the *CPPf* ratio.
